# Association of Vitamin D Concentrations with subjective health complaints in children and adolescents: the CASPIAN-V study

**DOI:** 10.1186/s12889-020-10020-z

**Published:** 2021-01-02

**Authors:** Nazli Namazi, Mostafa Qorbani, Gita Shafiee, Mohammad Hossein Ahmadian, Mohammad Esmaeil Motlagh, Mehdi Ebrahimi, Hamid Asayesh, Roya Kelishadi, Ramin Heshmat

**Affiliations:** 1 Chronic Diseases Research Center, Endocrinology and Metabolism Population Sciences Institute, Tehran University of Medical Sciences, Tehran, Iran; 2 Diabetes Research Center, Endocrinology and Metabolism Clinical Sciences Institute, Tehran University of Medical Sciences, Tehran, Iran; 3 Non-communicable Diseases Research Center, Alborz University of Medical Sciences, Karaj, Iran; 4 Department of Epidemiology, Endocrinology and Metabolism Research Center, Endocrinology and Metabolism Clinical Sciences Institute, Tehran University of Medical Sciences, Tehran, Iran; 5 Department of Pediatrics, Ahvaz Jundishapur University of Medical Sciences, Ahvaz, Iran; 6 Internal Medicine Department, Sina Hospital, Tehran University of Medical Sciences, Tehran, Iran; 7 Medical Emergencies, School of Paramedic, Qom University of Medical Sciences, Qom, Iran; 8 Child Department of Pediatrics, Child Growth and Development Research Center, Research Institute for Primordial Prevention of Non-communicable Disease, Isfahan University of Medical Sciences, Isfahan, Iran

**Keywords:** SHC, Vitamin D, School-aged children, CASPIAN

## Abstract

**Background:**

Vitamin D deficiency (VDD) is recognized as a global pandemic. Identification, any association between VDD and subjective health complaints (SHC), can be helpful to realize critical mechanisms and improve psychological and somatic symptoms. Given few studies published on this issue and the importance of its clarification, the main objective of this study was to examine the association between VDD and the SHC in children and adolescents.

**Methods:**

In this national cross-sectional study, 2596 Iranian children and adolescents aged 8–18 years were included. Data on SHC, anthropometric indices, physical activity, and serum levels of vitamin D were collected. Logistic regression models (crude, adjusted) were applied to examine the association between the VDD and the SHC. Statistical analysis was performed using STATA version 11. *P*-values< 0.05 were considered as statistically significant.

**Results:**

Serum levels of vitamin D in approximately 70% of Iranian children and adolescents were lower than 30 ng/mL. Among the SHC, irritability (40.9%) and feeling anxiety (33.7%) were the most prevalent ones. Multiple complaints in students with the VDD was 2.5 times greater than those with sufficient vitamin D concentrations (*p* <  0.001). Compared to the reference group, the strongest association was found between vitamin D status and difficulties in getting to sleep (OR: 2.5, 95%CI: 1.18, 3.53, p <  0.001).

**Conclusion:**

VDD was observed in the considerable percentage of the study population. There were no significant differences between the two gender groups. In addition, there were significant associations between vitamin D status and most of the somatic and psychological symptoms, particularly for getting to sleep. It seems national interventional programs for vitamin D supplementation or food fortifications can be helpful.

## Background

In the twenty-first century, vitamin D deficiency (VDD) is recognized as a global pandemic compared to other micronutrient deficiencies [1]. It has been estimated that more than 1 billion people across the world suffer from the VDD. The prevalence of the VDD in all age groups of men and women are remarkably increasing even in areas with sufficient sunlight, including the Middle East countries and North Africa [2]. Vitamin D status varies among societies and it depends on different factors including skin color, genetics, body weight, diet, geographical regions, and other environmental factors [3, 4].

Based on scientific evidence, vitamin D, a fat-soluble vitamin, plays pivotal roles in health maintenance [5]. Vitamin D not only contributes to calcium homeostasis and bone health, but also involves controlling metabolic parameters including blood pressure, glucose levels, the modulation of inflammatory parameters and immunity [2, 6]. Besides, vitamin D levels can affect the psychological health and behavioral factors [7, 8]. Earlier studies indicated that VDD could increase the rate of depression and anxiety in both children and adolescents [8, 9], but these findings are inconsistent.

To date, few studies have investigated the associations between Vitamin D status and any Subjective health complaints (SHC), and the results showed significant links [7, 8, 10, 11]. The SHC is a collection of somatic symptoms (e.g., headache, stomachache) and psychological symptoms (e.g., feeling nervous, depression) that cannot be justified by an underlying condition [12, 13]. It can also reflect the key dimensions of wellbeing. Health complaints are prevalent in children and adolescents, especially in girls and more than one symptom maybe reported at the same time by an individual [14, 15]. Health complaints can be associated with taking medicine, using the primary care services, and school absenteeism [16].

Identifying an association between the serum levels of vitamin D and SHC can be helpful to realize the critical mechanisms in the expression of psychological and somatic symptoms and reduce the severity of such health discomforts in school-age children using effective interventions. In general, Iran has moderate weather. However, some variations in type of weather are observed in various regions and sun exposure of children and adolescent is relatively low in this country. Although several studies have investigated the impacts of the VDD on the physical and psychological health of adults [17–20], very few studies have been conducted on its effects on children and adolescents. Given few studies on this issue and the importance of this link, the main objective of this study was to examine any associations between VDD and the SHC in children and adolescents.

## Methods

### Study design and participants

This national cross-sectional study was a part of the fifth survey of Childhood and Adolescence Surveillance and Prevention of Adult Non-communicable disease (CASPIAN)-V study in Iran (2014–2015). Details of the study protocol have been previously published [21]. Briefly, in a CASPIAN-V study, 14,400 students from both urban and rural are of thirty provinces of Iran were selected using a multi-stage, stratified cluster sampling in winter. In each province, considering the eligible criteria, sampling was conducted proportionally based on sex, the primary and secondary education level, and rural or urban residence location.

Only children and adolescents who met the following criteria were included in the study: (i) Iranian students at primary and secondary education level, (ii) having Iranian nationality, and (iii) no history of chronic diseases including confirmed cardiovascular diseases, diabetes, and cancers.

In each province, considering the eligibility criteria, sampling was conducted based on sex, primary and secondary education level, and rural or urban residence location.

For biochemical measurements, 14 clusters (10 subjects from each cluster) in each province were randomly chosen for biochemical analyses, and for this study, 2594 blood samples were selected for the assessment of serum Vitamin D concentrations.

Notably, after explaining the study objectives and its procedure, verbal and written informed consent was obtained from both students and their parents. Questionnaires, particularly for the younger, were filled out with the help of their parents. The Ethics committee of Isfahan University of Medical Sciences approved the study protocol (ID: 194049).

### Assessments

In the present study, the data on students’ basal characteristics, socio-economic status, screen time, anthropometric indices, physical activity, and the SHC were collected.

### Students’ basal characteristics

We used the questionnaire provided by the World Health Organization-Global School Student Health Survey (WHO-GSHS) to assess health behaviors and protective parameters related to the leading causes of morbidity and mortality of children and adolescents [22]. The validity and reliability of the Farsi format of the questionnaire were acceptable, as identified earlier (Cronbach’s alpha coefficient: 0.97, Pearson’s correlation coefficient: 0.94) [23].

### Socio-economic status

To estimate the SES of each student, five main parameters including parents’ educational level and occupation, type of school (public, private), home-ownership (yes, no), and family assets (having a personal computer, vehicle ownership) were considered. Finally, SES score was calculated as the weighted average of the mentioned items. Students were then classified into three groups (low, medium, and high SES).

### Screen time (ST)

To examine ST, participants were asked regarding the average time (hour/day) dedicated to watching TV and playing video games. After the calculation of total ST, students were classified into two groups (low and high). Those with less or equal to 2 h per day were placed in low; otherwise they were considered as individuals with high ST.

### Physical activity

A 7-day self-administrative physical activity questionnaire (PAQ-A) was used to examine the levels of physical activity. The PAQ-A is a valid and reliable questionnaire based on the information obtained previously (Cronbach’s alpha coefficient: 0.97, Pearson’s correlation coefficient: 0.94) [24].

### Anthropometric measurements

Body weight and the height of students were measured with standard methods by a trained member of the health care team as described before [21]. Body mass index (BMI) was calculated by dividing weight (kg) to the square of height (m).

### SHC

To assess the SHC, a valid questionnaire designed in the Health Behavior in School-aged Children (HBSC) study was applied [14]. The questionnaire was fulfilled using a face-to-face interview conducted with students. Participants were asked if they had experienced either any psychological (e.g., feeling low, feeling nervous, difficulty in getting sleep, irritability) or somatic symptoms (e.g., stomach ache, headache, backache, feeling dizzy) in the last six months before the study. The frequency of each sign was asked, as well.

Response options for each symptom were as follows: (i) about every day, (ii) more than once a week, (iii) about every week, (iv) about every month, (v) and (vi) rarely or never. Finally, the responses were categorized as “weekly or more” and “rarely or never.” All questionnaires are provided in **Supplementary files**.

### Biochemical assessments

To assess the serum levels of 25 (OH) D3, 6 mL of venous blood samples was taken from students. All samples were stored at − 70 °C until biochemical analysis.

To measure the serum concentration of 25-hydroxy vitamin D, a direct competitive immunoassay chemiluminescent method with LIASON 25-OH vitamin D assay TOTAL (DiaSorin,Inc.) was used (coefficient of variation: 9.8%).

Serum levels of vitamin D lower than 10 ng/mL (deficient), between 10 and 30 ng/mL (insufficient), and greater than 30 ng/mL (sufficient) were considered as deficient, insufficient, and sufficient, respectively.

### Statistical analysis

Qualitative and quantitative variables were expressed as percentages and means ± standard deviation (SD), respectively. Characteristics of students (such as age, location, SES, physical activity, and ST) and vitamin D levels in males and females were separately provided. The frequency of each complaint and multiple complaints related to health considering genders was presented as number and percentage. To assess the associations between vitamin D status and SHC, students were classified into three groups based on vitamin D concentrations (sufficient, insufficient, and deficient). The link between vitamin D status and SHC (8 health complaints) were examined across the mentioned three categories. In addition, the links that experienced multiple complaints were reported. Subjects with the sufficient levels of vitamin D3 (30–50 ng/mL) were considered as a reference group, and the two remaining groups (deficient, insufficient categories) were compared with this one for each SHC. Logistic regression models, both crude and adjusted models (3 models), were applied to examine any association. Covariates such as age, sex, region, SES, physical activity, ST, and BMI were adjusted in the mentioned adjusted models. Model 4 was controlled for all the above variables. Statistical analysis was performed using STATA version 11. *P*-values less than 0.05 were considered as statistically significant.

## Results

### Participants᾽ characteristics

In total, 2596 students (1166 girls and 1430 boys) were included in this study. The mean age of participants was 12.1 ± 3.0 years, and most participants (71.3%) were from urban areas. As presented in Table 1**,** there were significant differences between boys and girls with respect to all general characteristics of students except ST and rural or urban residence location. The sufficient vitamin D was observed in 29.0% of participants. There were no differences between boys and girls in terms of vitamin D concentrations (*p* = 0.18) (Table 1). Serum levels of vitamin D in approximately 70% of Iranian children and adolescents (7–18 years old) were lower than 30 ng/mL. However, no significant differences were found between the two gender groups.

**Table 1-:** General characteristics and vitamin D status in the study population (*n* = 2596)

Variables	Gender		*P*-value*
	Girl n (%)	Boy n (%)	
Age (years)			
7–12	672 (57.6)	748 (52.3)	0.007
13–18	494 (42.4)	682 (47.7)	
Location			
Urban	828 (71)	1022 (71.5)	0.798
Rural	338 (29)	408 (28.5)	
Socioeconomic status			
Poor	403 (36.6)	405 (29.4)	< 0.001
Good	331 (30.1)	496 (36)	
Well	366 (33.3)	477 (34.6)	
Physical activity			
High	461 (39.7)	647 (45.4)	0.004
Low	699 (60.3)	777 (54.6)	
Screen time			
≤ 2 h	985 (86.4)	1179 (84.8)	0.242
> 2 h	155 (13.6)	212 (15.2)	
Vitamin D Status (ng/mL)			0.182
< 10	132 (11.3)	142 (9.9)	
10–30	682 (58.5)	886 (62.0)	
> 30	352 (30.2)	402 (28.1)	

* Chi square for qualitative and t-test for quantitative variables

### Frequency of SHC

**Fig. 1:**
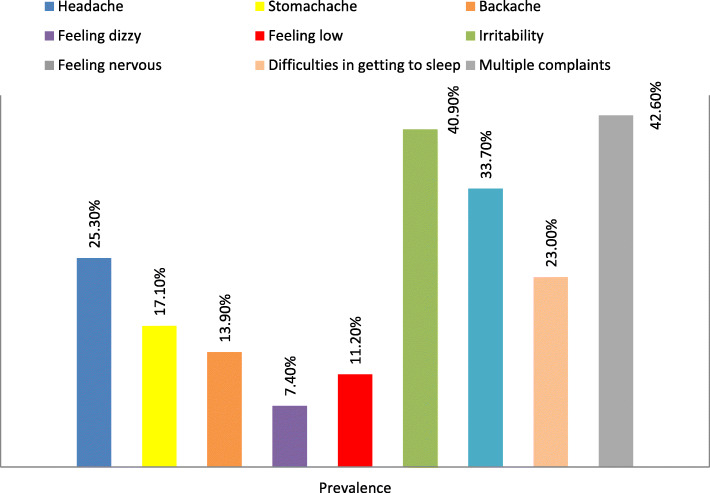
The prevalence of each subjective health complaint in the study populations

As presented in Fig. 1, the most prevalent health complaint was irritability (40.9%), which was followed by feeling anxiety (33.7%). Approximately 42.6% of participants experienced two or more complaints in the last 6 months (Fig. 1).

Table 2 shows the frequency of SHC in each gender category. A comparison of boys with girls revealed that in all complaints except feeling dizziness (*p* < 0.001), no significant differences existed. Girls experienced more dizziness than boys (9.4 vs.5.8%) in the last 6 months. In addition, no significant differences were found in the frequency of boys and girls with multiple health complaints (*p* = 0.27).

**Table 2:** The frequency of subjective health complaints based on gender

		Gender		*P*-value*
		Girl	Boy	
Headache n (%)	Yes	309 (26.5)	348 (24.4)	0.206
	No	856 (73.5)	1081 (75.6)	
Stomach ache n (%)	Yes	201 (17.3)	242 (17.0)	0.868
	No	964 (82.7)	1181 (83.0)	
Backache n (%)	Yes	163 (14.1)	192 (13.7)	0.805
	No	996 (85.9)	1207 (86.3)	
Feeling dizzy n (%)	Yes	109 (9.4)	81 (5.8)	< 0.001
	No	1046 (90.6)	1326 (94.2)	
Feeling low n (%)	Yes	131 (11.3)	156 (11.1)	0.872
	No	1027 (88.7)	1248 (88.9)	
Irritability n (%)	Yes	465 (40.1)	587 (41.6)	0.435
	No	696 (59.9)	825 (58.4)	
Feeling nervous n (%)	Yes	402 (34.6)	463 (32.9)	0.346
	No	759 (65.4)	946 (67.1)	
Difficulties in getting to sleep n (%)	Yes	278 (24.0)	311 (22.1)	0.259
	No	881 (76.0)	1096 (77.9)	
Multiple health complaints n (%)	Yes	511 (43.8)	596 (41.7)	0.271
	No	655 (56.2)	834 (58.3)	

*Chi square

### Frequency of SHC in vitamin D categories

As provided in Table 3**,** the frequency of subjects with all somatic and psychological symptoms except stomachache (*p* = 0.05) were significantly different among vitamin D categories. The occurrence of all remaining complaint except feeling dizziness in the deficient category was greater than insufficient and sufficient groups. The frequency of feeling dizziness in those with the serum levels of vitamin D between 10 and 30 ng/mL (8.5%) was higher than the other two categories. Also, the frequency of subjects with multiple health complaints in subjects with VDD (55.8%) was greater than the other two groups.

**Table 3:** Frequency of each subjective health complaints in each vitamin D classifications

Variables		Vitamin D status			*P* value*
		Deficient	Insufficient	Sufficient	
Headache n (%)	Yes	88 (32.1)	404 (25.8)	165 (21.9)	0.003
	No	186 (67.9)	1163 (74.2)	588 (78.1)	
Stomachache n (%)	Yes	57 20.8	275 17.6	111 14.8	0.054
	No	217 (79.2)	1287 (82.4)	641 (85.2)	
Backache n (%)	Yes	55 20.4	213 13.8	87 11.7	0.002
	No	214 79.6	1331 86.2	658 88.3	
Feeling dizzy n (%)	Yes	18 (6.7)	132 (8.5)	40 (5.4)	0.025
	No	249 (93.3)	1419 (91.5)	704 (94.6)	
Feeling low n (%)	Yes	42 (15.5)	179 (11.6)	66 (8.9)	0.010
	No	229 (84.5)	1368 (88.4)	678 (91.1)	
Irritability n (%)	Yes	130 (47.8)	660 (42.6)	262 (34.9)	< 0.001
	No	142 (52.2)	891 (57.4)	488 (65.1)	
Feeling nervous n (%)	Yes	118 (43.9)	519 (33.4)	228 (30.6)	< 0.001
	No	151 (56.1)	1036 (66.6)	518 (69.4)	
Difficulties in getting to sleep n (%)	Yes	91 (33.6)	367 (23.7)	131 17.5%	< 0.001
	No	180 (66.4)	1179 (76.3)	618 (82.5)	
Multiple health complaints n (%)	Yes	153 (55.8)	697 (44.5)	257 (34.1)	< 0.001
	No	121 (44.2)	871 (55.5)	497 (65.9)	

*Chi square

### The association between VDD and SHC

Multivariable-adjusted odds ratio (OR) for VDD and each health complaint is reported in Table 4. The sufficient vitamin D category was considered as a reference group and the other two groups were compared with this one. Both crude (model 1) and adjusted models (models 2–4) are provided in Table 4.

**Table 4:** The associations between subjective health complaints with vitamin D status

	Variables	Vitamin D level				
		Sufficient (reference)	Deficient OR (95% CI)	*P*-value*	insufficient OR (95% CI)	*P*-value*
Headache	Model 1	1	1.686 (1.241–2.291)	0.001	1.238 (1.007–1.522)	0.043
	Model 2	1	1.667 (1.226–2.268)	0.001	1.240 (1.008–1.526)	0.042
	Model 3	1	1.636 (1.186–2.256)	0.003	1.182 (0.953–1.464)	0.128
	Model 4	1	1.653 (1.197–2.282)	0.002	1.183 (0.955–1.467)	0.124
Stomachache	Model 1	1	1.517 (1.064–2.162)	0.021	1.234 (0.971–1.568)	0.086
	Model 2	1	1.510 (1.059–2.154)	0.023	1.236 (0.972–1.572)	0.084
	Model 3	1	1.626 (1.127–2.347)	0.009	1.189 (0.928–1.525)	0.171
	Model 4	1	1.646 (1.139–2.377)	0.008	1.191 (0.929–1.528)	0.167
Backache	Model 1	1	1.944 (1.341–2.818)	< 0.001	1.210 (0.927–1.580)	0.160
	Model 2	1	1.936 (1.334–2.811)	0.001	1.213 (0.928–1.584)	0.157
	Model 3	1	2.016 (1.359–2.991)	< 0.001	1.209 (0.914–1.599)	0.184
	Model 4	1	2.038 (1.373–3.025)	< 0.001	1.211 (0.916–1.603)	0.179
Feeling dizzy	Model 1	1	1.272 (0.716–2.260)	0.412	1.637 (1.136–2.359)	0.008
	Model 2	1	1.269 (0.713–2.261)	0.418	1.682 (1.166–2.428)	0.005
	Model 3	1	1.432 (0.785–2.610)	0.242	1.849 (1.262–2.709)	0.002
	Model 4	1	1.455 (0.797–2.655)	0.222	1.850 (1.263–2.710)	0.002
Feeling low	Model 1	1	1.884 (1.244–2.853)	0.003	1.344 (0.999–1.809)	0.051
	Model 2	1	1.852 (1.220–2.814)	0.004	1.341 (0.995–1.808)	0.054
	Model 3	1	1.931 (1.240–3.007)	0.004	1.323 (0.967–1.810)	0.080
	Model 4	1	2.006 (1.285–3.131)	0.002	1.332 (0.973–1.822)	0.073
Irritability	Model 1	1	1.705 (1.287–2.259)	< 0.001	1.380 (1.152–1.653)	< 0.001
	Model 2	1	1.701 (1.282–2.257)	< 0.001	1.381 (1.151–1.656)	< 0.001
	Model 3	1	1.695 (1.259–2.282)	0.001	1.341 (1.109–1.622)	0.002
	Model 4	1	1.727 (1.282–2.328)	< 0.001	1.346 (1.113–1.628)	0.002
Feeling nervous	Model 1	1	1.775 (1.333–2.365)	< 0.001	1.138 (0.943–1.374)	0.177
	Model 2	1	1.761 (1.320–2.349)	< 0.001	1.139 (0.943–1.376)	0.176
	Model 3	1	1.682 (1.242–2.278)	0.001	1.148 (0.942–1.399)	0.172
	Model 4	1	1.729 (1.275–2.344)	< 0.001	1.153 (0.945–1.405)	0.160
Difficulties in getting to sleep	Model 1	1	2.385 (1.741–3.267)	< 0.001	1.468 (1.176–1.833)	0.001
	Model 2	1	2.362 (1.722–3.239)	< 0.001	1.470 (1.176–1.837)	0.001
	Model 3	1	2.516 (1.803–3.512)	< 0.001	1.535 (1.213–1.942)	< 0.001
	Model 4	1	2.532 (1.813–3.537)	< 0.001	1.536 (1.214–1.944)	< 0.001
Multiple complaints	Model 1	1	2.445 (1.844–3.242)	< 0.001	1.548 (1.292–1.854)	< 0.001
	Model 2	1	2.436 (1.834–3.236)	< 0.001	1.559 (1.300–1.870)	< 0.001
	Model 3	1	2.526 (1.875–3.402)	< 0.001	1.577 (1.304–1.907)	< 0.001
	Model 4	1	2.557 (1.897–3.447)	< 0.001	1.580 (1.307–1.911)	< 0.001

*Logistic Regression; *OR* Odd Ratio, *CI* Confidence Intervals

Model 1: Crude; Model 2: adjusted for age, sex and residential region; Model 3: Model 2+ socio-economical status, physical activity, and screen time; Model 4: Model 3+ body mass index

In crude models, children and adolescents with VDD experienced all complaints except sensation of dizziness (OR: 1.27, 95%CI: 0.71, 2.26, *p* = 0.41) more than those with the sufficient levels of vitamin D. After controlling for confounders including age, sex, physical activity, ST, and BMI, all associations remained significant. In addition, multiple complaints in students with VDD was 2.5 times greater than those with sufficient vitamin D concentrations (*p* < 0.001). Compared to the reference group, the strongest association was found between VDD and difficulties in getting to sleep. No considerable changes were observed after controlling for confounders (OR: 2.5, 95%CI: 1.18, 3.53, *p* < 0.001).

Comparison between students with insufficient levels of vitamin D and those with sufficient levels showed that the insufficiency of vitamin D was positively associated with headache, feeling dizzy, irritability, and difficulties in getting to sleep. After controlling confounders, there was no link between headache and vitamin D status (*p* = 0.12). Similar to the VDD category, the strongest relationship was observed between difficulties in getting to sleep and insufficient levels of vitamin D. However, in this category, the link was weaker than the VDD category (OR: 1.53 vs. 2.53).

## Discussion

Based on this cross-sectional study, serum levels of vitamin D in approximately 70% of Iranian children and adolescents were lower than 30 ng/mL. In addition, there were significant associations between vitamin D status and somatic and psychological symptoms in school-age children. Among SHC items, difficulties in getting to sleep showed the strongest association with VDD and the insufficient levels of vitamin D compared to sufficient levels.

A systematic review and meta-analysis (2018) on Iranian studies revealed that 35 and 61% of Iranian boys and girls suffered from VDD, respectively. Based on their estimation, the prevalence of vitamin D insufficiency in Iranian children and adolescents was 31% [25]. In their study, Turer et al. indicated that the prevalence rates of VDD (defined as 25-hydroxyvitamin-D < 20 ng/mL) in healthy-weight and obese American children aged 6–18 years were 21 and 34%, respectively [26]. Al-Shaikh et al. reported that 95.3% of Saudi children (6–15 years old) had either Vitamin D insufficiency (49.9%) or VDD (45.5%) [27]. According to a study conducted by Shin et al., vitamin D insufficiency or VDD was found in 98.9% of Korean boys and 100% of girls [28]. Sahu et al. also revealed that despite much sunlight in India, VDD (< 50 nmol/L) in Indian adolescent girls was 88.6% [29]. Differences in the cut-off points for vitamin D status, age range, seasonal variation, genetic, geographical, and environmental differences can affect findings. Some studies, for instance, examined dietary intake of vitamin D in Iran and reached insufficient intake [30, 31]. Mean daily dietary intake of Vitamin D was 0.54 μg/day in the Southwest of Iran, Khuzestan, which was less than standard recommended level [30, 31]. In another study in the capital of Iran, Tehran, the mean intake of vitamin D from food products was 100 IU/day [30]. Regarding the effects of genetic on serum levels of vitamin D and its associations with diseases, several studies showed the effects of related gene polymorphisms in the occurrence of diseases in Iran [32–34].

To the best of our knowledge, this is the first national survey in developing countries the examining association between vitamin D status and SHC in children and adolescents.

In the present study, 55.8% of subjects with VDD reported multiple health complaints. Irritability (47.8%), feeling nervous (43.9%), and difficulties in getting to sleep (33.6%) were the most frequent complaints among the study population. Our findings were consistent with those of Ataie et al.’s study suggesting that some psychological distress, including anxiety, anger, depression, poor quality sleep, and worry were associated with hypovitaminosis D in Iranian adolescents [35].

Pathways through which vitamin D affects mental health have not been fully understood yet. However, some possible mechanisms have been proposed. Vitamin D is a neurosteroid hormone that can regulate the metabolism of neurotransmitters in the central nervous system [36]. Vitamin D can affect monoamine neurotransmitters, including norepinephrine and serotonin that play remarkable roles in depression and mood disorders [37, 38]. In addition, in the central nervous system, vitamin D receptors, 25(OH) D 1-α-hydroxylase, and the cytochrome P-450 that catalyze the hydroxylation of calcidiol to calcitriol, the active form of vitamin D, have been reported [39].

Furthermore, studies on sleep–wake regulation and vitamin D target neurons showed positive impacts of vitamin D on sleep [40]. In a systematic review and meta-analysis, Gao et al. showed that the VDD increased sleep disorders by 50%. Serum levels of 25(OH) D less than 20 ng/mL can enhance the risk for unhealthy sleep. They also revealed that VDD was associated with poor sleep quality and short sleep duration [41]. Vitamin D receptors are existed in the central nervous system and their distribution in several parts of the brain including the hypothalamus, midbrain central gray, and prefrontal cortex are likely to affect sleep regulation [41].

This study proves that there were significant associations between vitamin D status and most somatic and psychological symptoms. However, genetic differences, dietary habits, taking supplements, and direct exposure to sunlight need to be examined in the future prospective cohort studies in order to clarify a cause and effect relationship between VDD and SHC in children and adolescents.

Notably, Iran’s climate is diverse and it has 11 climates out of 13 in the world. It ranged from arid and semi-arid, to subtropical along the Caspian coast and the northern forests. This study was a national survey and subjects were from different regions of Iran. In all regions there are specific places for indoor activities for children and adolescents. As the study was in winter, sun exposure was low in all regions of Iran.

This study has some limitations that should be pointed out. Firstly, due to the cross-sectional design of the study, we could not clarify any cause and effect relationships between vitamin D status and SHC. Secondly, we did not consider dietary intake and taking vitamin D supplement in the study population. Thirdly, the data related to puberty were not collected. It should be kept in our mind that in the present study girls contained about 45% of the study population and were either in the pre-menarche or had already started menstruation. A lot of SHC symptoms can overlap with symptoms associated with menstruation and might have an effect on Vitamin D levels or vice versa. Fourthly, genetic assessments like the determination of polymorphisms were not performed. However, this is the first national survey addressing this topic and provides a ground for interventional studies through the identification of VDD and SHC prevalence among school-age children. The results are helpful for both policymakers and clinicians and they clarified the necessity of national interventional programs for vitamin D supplementation or food fortifications with vitamin D in Iran.

## Conclusion

This study demonstrated that there were significant associations between vitamin D status and the somatic and psychological symptoms. Among SHC items, difficulties in getting to sleep showed the strongest relationship with vitamin status. VDD increased the experience of difficulties in getting to sleep by 2.5 times compared to a sufficient level.

## Supplementary Information

**Additional file 1.** Questionnaires-SHC.

## Data Availability

The source of the raw data analyzed in this study is a national survey named CASPIAN study. They are available and can be provided (by Dr. Mostafa Qorbani) with reasonable reason. However, the dataset supporting the conclusions of this paper will not be shared publicly, to ensure participants’ privacy.

## Abbreviations

VDD: Vitamin D deficiency
SHC: Subjective Health Complaints
CASPIAN-V: Fifth survey of Childhood and Adolescence Surveillance and Prevention of Adult Non-communicable disease
WHO-GSHS: World Health Organization-Global School Student Health Survey
SES: Socio-economic status
ST: Screen time
PAQ: Physical activity questionnaire
BMI: Body mass index
HBSC: Health Behavior in School-aged Children
OR: Odds ratios
