# Supplementary figures and images for: Association of Vitamin D Concentrations with subjective health complaints in children and adolescents: the CASPIAN-V study
Source: BMC Public Health. 2021 Jan 2;21:3. doi: 10.1186/s12889-020-10020-z (PMC7778822; doi:10.1186/s12889-020-10020-z)

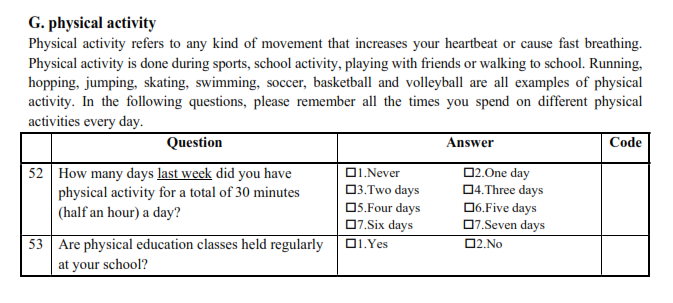


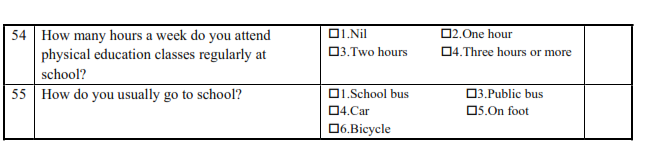


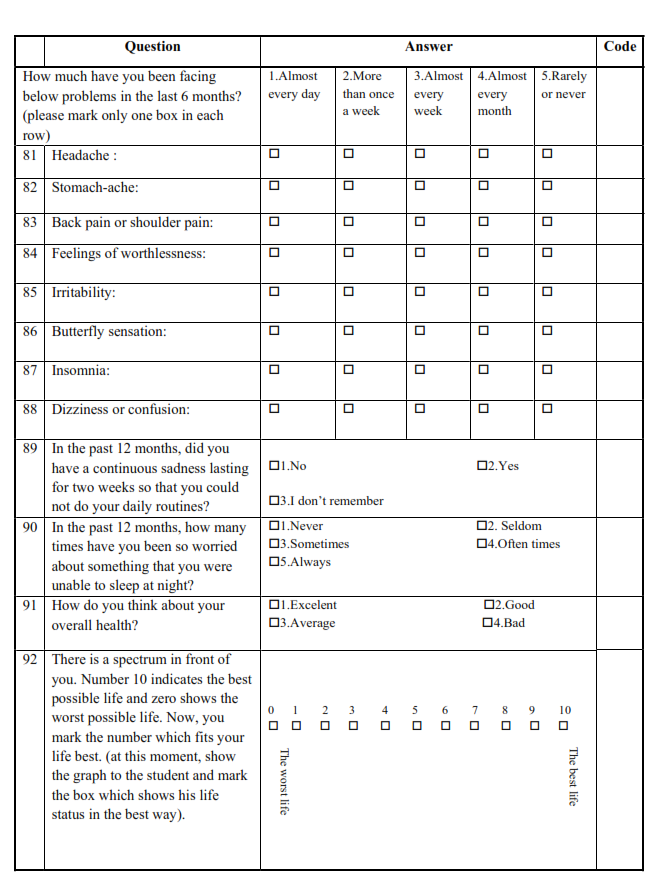


**Subjective Health Complaints Questionnaire**


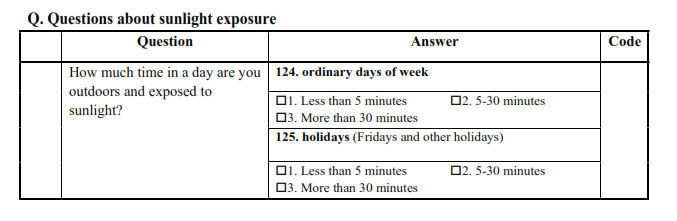


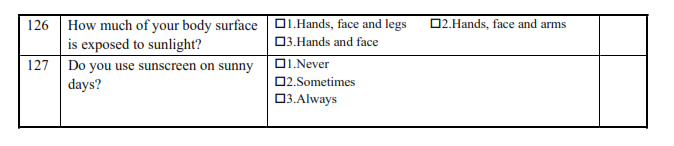


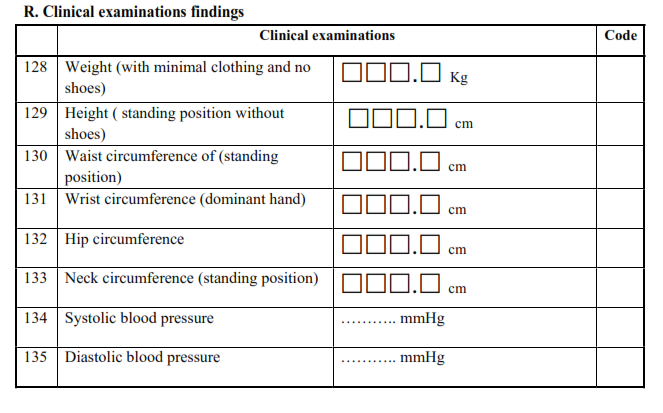


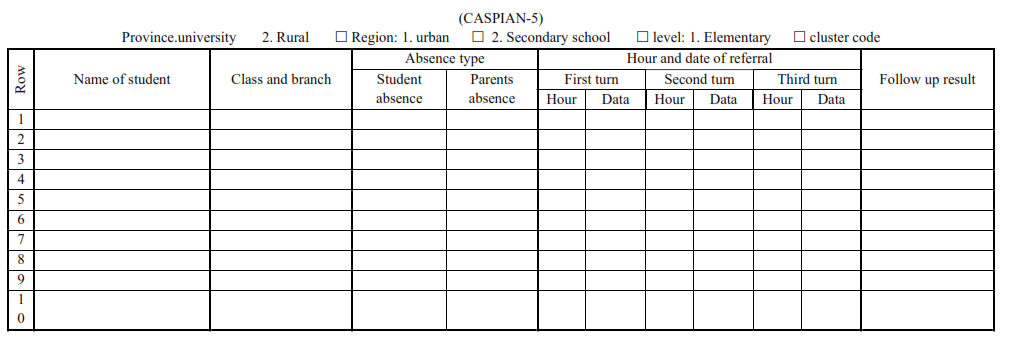

Supplement: Supplementary file 1 — Additional file 1. Questionnaires-SHC. [file 12889_2020_10020_MOESM1_ESM.docx]
